# PlGF Immunological Impact during Pregnancy

**DOI:** 10.3390/ijms21228714

**Published:** 2020-11-18

**Authors:** Loredana Albonici, Monica Benvenuto, Chiara Focaccetti, Loredana Cifaldi, Martino Tony Miele, Federica Limana, Vittorio Manzari, Roberto Bei

**Affiliations:** 1 Department of Clinical Sciences and Translational Medicine, University of Rome “Tor Vergata”, Via Montpellier 1, 00133 Rome, Italy; monica.benvenuto@unicamillus.org (M.B.); chiara.focaccetti@uniroma5.it (C.F.); cifaldi@med.uniroma2.it (L.C.); manzari@med.uniroma2.it (V.M.); bei@med.uniroma2.it (R.B.); 2 Saint Camillus International University of Health and Medical Sciences, Via di Sant’ Alessandro 8, 00131 Rome, Italy; 3 Department of Human Science and Promotion of the Quality of Life, San Raffaele Roma Open University, Via di Val Cannuta 247, 00166 Rome, Italy; federica.limana@uniroma5.it; 4 Academic Department of Pediatrics (DPUO), Ospedale Pediatrico Bambino Gesù, IRCCS, Piazza Sant’ Onofrio 4, 00165 Rome, Italy; 5 Department of Experimental Medicine, University of Rome “Tor Vergata”, Via Montpellier 1, 00133 Rome, Italy; miele@med.uniroma2.it; 6 Laboratory of Cellular and Molecular Pathology, IRCCS San Raffaele Pisana, Via della Pisana 235, 00163 Rome, Italy

**Keywords:** PlGF, Flt-1/VEGFR1, pregnancy, immune modulation

## Abstract

During pregnancy, the mother’s immune system has to tolerate the persistence of paternal alloantigens without affecting the anti-infectious immune response. Consequently, several mechanisms aimed at preventing allograft rejection, occur during a pregnancy. In fact, the early stages of pregnancy are characterized by the correct balance between inflammation and immune tolerance, in which proinflammatory cytokines contribute to both the remodeling of tissues and to neo-angiogenesis, thus, favoring the correct embryo implantation. In addition to the creation of a microenvironment able to support both immunological privilege and angiogenesis, the trophoblast invades normal tissues by sharing the same behavior of invasive tumors. Next, the activation of an immunosuppressive phase, characterized by an increase in the number of regulatory T (Treg) cells prevents excessive inflammation and avoids fetal immuno-mediated rejection. When these changes do not occur or occur incompletely, early pregnancy failure follows. All these events are characterized by an increase in different growth factors and cytokines, among which one of the most important is the angiogenic growth factor, namely placental growth factor (PlGF). PlGF is initially isolated from the human placenta. It is upregulated during both pregnancy and inflammation. In this review, we summarize current knowledge on the immunomodulatory effects of PlGF during pregnancy, warranting that both innate and adaptive immune cells properly support the early events of implantation and placental development. Furthermore, we highlight how an alteration of the immune response, associated with PlGF imbalance, can induce a hypertensive state and lead to the pre-eclampsia (PE).

## 1. Introduction

### 1.1. The Immunological Paradox of Human Pregnancy

In the middle of the last century, Peter Medawar was the first to question the complexity of the immunological networks that allow maternal-fetal tolerance. Although Medwar’s intuition was still far from the description of the real immune mechanisms, the immunological tolerance, especially during the early stages of pregnancy, represented a current issue that has only been partially clarified [1].

**Figure 1:**
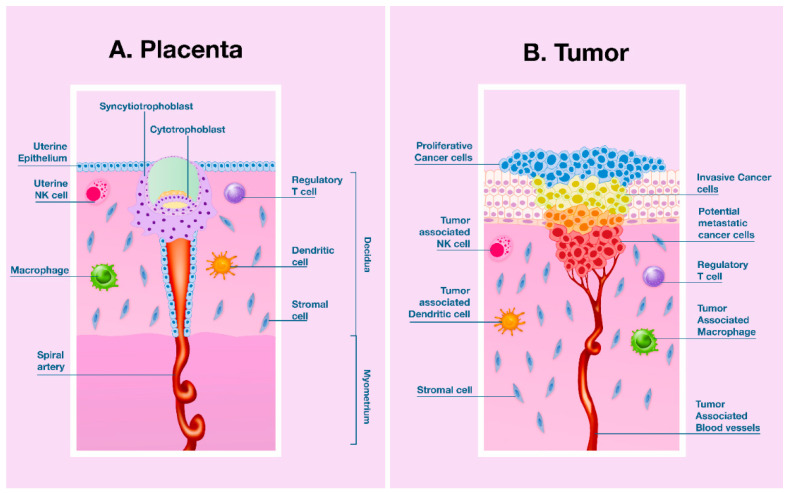
Human placentation and malignancy share proliferative and invasive features to establish a nutrient supply and evade or modify the host’s immune response. (**A**) Human placenta development. In the early stage of implantation, the blastocyst displays an invasive phenotype that allows implanting inside the endometrial stroma. In this process cytokines, growth factors, hormones, extracellular matrix metalloproteinases (MMPs), and immune cells, all modulate cell invasion of maternal decidua and myometrium and their capacity to transform spiral arteries. Among growth factors, placental growth factor (PlGF) secreted by the decidua, trophoblast, and uterine natural killer (uNK) cells have a determinant role in regulating invasion, vascular development, and maternal immune tolerance mechanisms to semi-allogeneic fetus; (**B**) Tumor invasion and progression. Malignancy is able to create both a vascular network that warrants perfusion of tumor mass and an immunosuppressive microenvironment by recruiting specific immune cells. Molecules (cytokines, growth factors, extracellular MMPs), produced by tumor and inflammatory cells in the tumor microenvironment, recruit (tumor-associated) immune cells, thus, creating a tolerogenic milieu that inhibits the development of an efficient immune response against cancer cells that foster tumor growth and progression. PlGF blockade reduces both neoangiogenesis and lymphangiogenesis, inhibits the M2 macrophages polarization, hinders the recruitment of tumor-associated macrophages (TAM), and decreases the recruitment of myeloid suppressor cells [23]. (Illustration inspired by Holtan et al. [21] with kind permission of Elsevier, License Number: 4911830648497).

The uterine microenvironment can be considered to be a privileged immune site similar to other body districts (e.g., mucosae-associated lymphoid tissue), where the immune response is tightly controlled to prevent improper activation of immune cells. However, recent evidence supports the idea that the immune response, at the maternal-fetal interface, is not suppressed, and instead, is in a highly dynamic state [2,3,4]. The placenta develops from the outer cell layer of the blastocyst, which forms the primary trophoblastic cell mass. Then, the trophoblast, which represents the fetal part of placenta and contains the father’s genetic material, deeply invades the decidua and usually its development is not counteracted by the maternal immune system [5,6,7,8,9,10]. Indeed, implantation implicates attachment and invasion of the blastocyst into the uterus and remodeling of the endometrial stroma, known as “decidualization”, as well as modifications of the endometrial vascular bed. Thus, the implantation site is characterized by an inflammatory response, with an abundance of recruited immune cells and the induction of inflammatory genes [11,12,13,14,15]. In addition, the blastocyst needs to aggressively adhere to the endometrium to obtain oxygen and nutrients. During this process, uterine tissue remodeling and inflammatory mechanisms are required [4,16,17,18]. This proliferative and invasive behavior, associated with immune tolerance mechanisms, is similar to that activated by invasive cancer cells, for obtaining a nutrient supply and evading or editing a host immune response [19,20,21,22]. Therefore, in addition to the ability to invade normal tissues, both developing placenta cells and cancer cells are able to create a microenvironment supportive of angiogenesis and immunological privilege [13,21,22,23,24,25] (Figure 1). Although these two processes show overlapping mechanisms, pregnancy is still a physiological process unlike tumor invasion, which is a pathological process. Consequently, a fine balance between inflammatory and anti-inflammatory mediators is required to prevent fetal rejection [7,13,26]. Among the several inflammatory mediators present in the uterus, a predominant role is played by placental growth factor (PlGF) [12,27,28,29,30,31,32]. In addition to being a factor involved in the implantation and development of the placenta, PlGF also has an impact on the immune response by acting on both innate and adaptive immune cells [23,29,33,34,35,36,37].

Although numerous authors have attributed the onset of pre-eclampsia (PE) to maternal cardiovascular system maladaptation [38,39,40,41,42], the idea that PE is a pathological condition associated with an alteration of the vascular development of the placenta [43] and it is also the result of an aberrant maternal immune response has become increasingly consistent [44,45,46,47,48,49]. This hypothesis stems from the evidence that immune-mediated mechanisms are able to regulate the cells response to angiogenic growth factors and, in turn, angiogenic growth factors can modulate the behavior of immune cells. In addition, angiogenic growth factor PlGF imbalance has long been associated with pathological pregnancies, such as implantation failure and the development of PE [48,49,50,51,52,53,54,55,56,57,58,59,60]. In this review, we discuss how an altered maternal immune response, modulated by PlGF imbalance, can induce a hypertensive state and lead to pathological pregnancy.

### 1.2. Placental Growth Factor (PlGF)

PlGF is a pleiotropic angiogenic growth factor originally isolated from the human placenta in which it displays a major role in vasculogenesis and angiogenesis [61,62]. It is encoded by a single *plgf* gene belonging to the vascular endothelial growth factor (VEGF) family and gives rise to four splice isoforms in humans, PlGF-1–4 [61]. Whereas PlGF-1 and PlGF-3 are diffusible isoforms, PlGF-2 and PlGF-4 have heparin-binding domains. Mice express only one isoform equivalent to human PlGF-2. Heparin can modulate PlGF-2-induced proliferation of extravillous trophoblast (EVT) cells, by sequestering PlGF-2 from its receptor or by modifying its structural conformation, without affecting migration or invasiveness of EVT cells [28].

PlGF expression is low to undetectable in most tissues in normal health; however, its angiogenic activity is mainly restricted to pathological conditions, such as inflammation or ischemia [12,63,64,65]. PlGF is upregulated by different stimuli such as hypoxia, inflammatory cytokines, growth factors, and hormones, all events present during implantation and placenta development. Hypoxia also upregulates the PlGF receptor fms-related tyrosine kinase (Flt)-1/vascular endothelial growth factor receptor (VEGFR)-1 and its co-receptor neuropilin-1 (NRP1) in disease conditions [66,67,68,69]. However, unlike tumor growth, during pregnancy, the transcriptional activity of PlGF is suppressed by hypoxia and increased by a normoxic environment in the trophoblast, indicating a specific regulatory mechanism in these cells. Furthermore, the regulation of PlGF transcription in trophoblast cells is not mediated by the functional activity of hypoxia-inducible factor (HIF)-1 [70]. Since PlGF plays an important role in the vascular development of the placenta, these results suggest the existence of a protective regulation mechanism of PlGF levels in the prevention of the oxidative damage caused by hypoxia. PlGF has been shown to play a negligible role in the development and in the physiological angiogenesis process, even if adult *plgf^−/−^* mice are apparently healthy, fertile, and without vascular defects [71]. Nevertheless, in mice with genetic deletion of *plgf,* uterine natural killer (uNK) cells were smaller, less granular, and binucleated than in control mice. However, uNK cell numbers were almost unchanged as compared with control mice, indicating that PlGF plays an important role in successful uNK cells proliferation or differentiation [72]. However, although decidual invasion was not influenced in *plgf^−/−^* mice, the implantation site showed abnormal placental vasculature with decreased branching of feto-placental vessels and increased lacunarity, indicating a lack of uniformity of vessel formation [73].

During pregnancy, the placental trophoblast is the main source of PlGF and its expression is significantly upregulated at an early gestational age after implantation [12,27,28,74]. PlGF is also produced by the human endometrium and released into the uterine lumen [29]. An additional source of PlGF during implantation is from the production mediated by uNK cells [30,75]. Accordingly, the abnormal expression of PlGF during pregnancy affects the trophoblast function as much as the vascularity in the placental bed [30,53,75,76,77]. Immunohistochemical analysis has shown that PlGF was significantly lower in the placentas of women with severe PE as compared with those with mild PE and placentas of normotensive women [52]. This result confirmed previous data from serum levels of PlGF. Indeed, serum PlGF levels were lower among women who developed PE (23 ± 24 pg/mL vs. 63 ± 145 pg/mL) or gestational hypertension (27 ± 19 pg/mL) as compared with the controls [60].

PlGF homodimers bind Flt-1/VEGFR-1 [67]. However, only PlGF-2 and PlGF-4 are able to bind the co-receptors NRP1 and NRP2, due to the insertion of 21 basic amino acids [78,79,80]. Although the downstream Flt-1/VEGFR-1 signaling is still elusive under physiological condition, Flt-1/VEGFR-1 plays a negative role by suppressing pro-angiogenic signals, as displayed by an early death in embryogenesis due to the uncontrolled growth of endothelial cells and disorganization of the vascular architecture in *vegfr1-null* mutant mice [81].

In addition to the direct effects on endothelial cells, the binding of PlGF to Flt-1/VEGFR-1 shows indirect effects on nonvascular cells by modulating the behavior of immune cells [23,71,82,83,84,85,86,87]. PlGF enhances macrophages proliferation, migration, and survival [33] and also shows a direct effect on the inflammatory reaction by triggering tumor necrosis factor (TNF)-α and interleukin (IL)-6 production in a calcineurin-dependent pathway [86]. PlGF significantly increases IL-8 secretion, cyclooxygenase (Cox)-2 expression, and consequent prostaglandin (PG)-E2 and PG-F2α release, matrix metalloproteinases (MMP)-9 gene expression, and enzyme production via Flt1/VEGFR1 on monocytes [35,36]. Overall, all these molecules play a role during the decidualization and tumor cell growth and progression (Figure 1). Moreover, TNF-α, by promoting PlGF synthesis, can regulate angiogenesis via PlGF/Flt-1/VEGFR-1 [87]. Flt-1/VEGFR-1 is a cell surface marker for the monocyte-macrophage lineage in humans [85], and it is also expressed on the surface of activated T cells in which it increases migration and IL-10 production [88], thus, indicating that Flt-1/VEGFR-1 is able to mediate a direct immunomodulatory effect.

The co-receptors NRP1 and NRP2 were initially characterized as receptors expressed by neuronal cells, where the natural ligand of NRP1 was semaphoring 3A (Sema3A) and, subsequently, endothelial cells [89]. Apart from vessels and axons, NRPs are also expressed by immune cells in which they display an inhibitory effect [90]. NRP1 is expressed primarily by dendritic cells (DCs) and regulatory T (Treg) cells [66,91] and exerts mainly inhibitory effects on the immune response. Indeed, NRP1 is constitutively expressed at a high level, independently of its activation status, only on the surface of CD4^+^CD25^high^ natural Treg cells (nTreg), which arise in the thymus, but not on inducible Treg cells (iTreg) generated in the periphery through the induction of Foxp3 [92]. NRP1 functions as a component of the immunological synapse and promotes prolonged interaction between Treg cells and immature dendritic cells (iDCs). This long contact results in higher nuclear factor kappa beta (NF-*κ*B) transcriptional activity that might prevent an autoimmune response by inducing immunosuppression probably because of the delay of iDCs maturation [93]. These findings could envisage a possible competition between PlGF and Sema3A as they bind NRP1 in the same domain and, consequently, an immunosuppressive role played by PlGF. Although NRP1 downregulation has been rarely described in few studies involving PE or fetal growth restriction due to deficient vascular branching [94,95], a recent study by Moldenhauer et al. confirmed the role of NRP1 on the immune system during the preimplantation period. They reported that mating mice elicited a five-fold increase in uterine Treg cells, followed by an extensive Treg proliferation in the uterus-draining lymph nodes, comprising 70% NRP1^+^ thymus-derived Treg cells and 30% NRP1^−^ peripheral Treg cells, as compared with virgin mice. This increase was due to epigenetic modification of the transcription factor Foxp3, induced by the presence of the seminal fluid [96].

The alternative splicing of both Flt1/VEGFR-1 and NRP1 pre-mRNA produces soluble receptor isoforms (sFlt-1/sVEGFR-1 and sNRP1, respectively) that can bind to and inhibit the action of both PlGF and VEGF [97,98,99]. Excessive sFlt-1/sVEGFR-1 generated by the human placenta and released into the maternal circulation leads to hypertension and proteinuria in PE, thus, contributing to maternal vascular injury [50,51,53,55,56,59,77,100,101,102,103,104]. At least four different tissue-specific splice variants of sFlt-1/sVEGFR-1 have been described [101,105,106]. Among these variants, sFlt-1/sVEGFR-1 e15a is the main variant produced primarily in the placenta and it is believed to be responsible for PE, being significantly elevated in the placenta and circulation of women with PE as compared with normal pregnancies. sFlt-1/sVEGFR-1 e15a could be responsible for endothelial dysfunction and terminal organ dysfunction as observed in PE-like mouse models. These biochemical changes appear to precede the clinical features of disease [106].

## 2. Pregnancy and Inflammation

The inflammatory response of the PlGF-Flt-1/VEGFR-1 axis is mediated by the transcription factor NF-*κ*B involved in genes related to inflammation and immune response regulation. NF-*κ*B has regulatory binding sites in the promoter of the PlGF gene and can modulate the transcriptional activity of PlGF via Rel-A in hypoxic human monocytes [107,108]. PlGF, by increasing the degradation of I*κ*B-α, can increase the DNA binding activity of p65, an NF-*κ*B subunit, thus, generating a self-feeding mechanism [107]. NF-*κ*B is involved in several cellular pathways including inflammation, hypoxia, and angiogenesis, all processes implicated in placental development, thus consequently, when dysregulated, it is considered to be one of the main factors responsible for PE [109,110,111]. In fact, NF-*κ*B is involved in the production of both TNF-α and PlGF resulting in aberrant activation of innate immune cells and imbalanced differentiation of CD4^+^ T lymphocyte subsets, which may account for high cytokine levels and the cytotoxic environment in utero [48,112,113,114].

In normal pregnancy, three different phases can be identified, each characterized by a specific correlation between NF-*κ*B and PlGF. The first trimester is characterized by a proinflammatory profile in which NF-*κ*B is activated by inflammation and hypoxia that occurs during the development of the placenta. The second trimester shows an anti-inflammatory state as the pregnancy progresses. During the third trimester, NF-*κ*B returns to be expressed at high levels in the decidua in preparation for parturition [115]. In addition, NF-*κ*B can also directly upregulate the expression of TNF-α by lymphocytes during hypoxic stress, thus, establishing a vicious circle that feeds inflammation [116,117]. Therefore, the expression of NF-*κ*B is critical in maintaining an adequate level of cytokines/PlGF required during the different periods of pregnancy.

A further link between the immune response and PlGF results from the involvement of another transcription factor, namely nuclear factor of activated T cells (NFAT)-1, which was initially identified in activated T cells [118,119] and has also been shown to be involved in the control of innate immunity [120]. Cytoplasmic NFAT is activated through calcineurin-mediated dephosphorylation, then, NFAT translocates into the nucleus, where it upregulates the expression of IL-2 and stimulates the growth and differentiation of the T cells [118]. Ding et al. reported that TNF-α was upregulated by tumor-derived PlGF in myelomonocytic cells via NFAT-1, which in turn contributed to the recruitment of PlGF-induced myelomonocytic cells [34]. Moreover, a region of the Flt-1/VEGFR-1 promoter contains a binding site for the transcription factor NFAT-1, thus, providing evidence that Flt-1/VEGFR-1 represents a NFAT-1 target gene [121]. The definitive confirmation of the role played by the PlGF-Flt-1/VEGFR-1-NFAT-1 axis in the placenta derives from the evidence that the inhibition of NFAT reduces both Flt-1/VEGFR-1 and sFlt-1/sVEGFR-1 splice variant e15a transcript secretion from primary human cytotrophoblasts [122]. In addition to being involved in cell proliferation, invasive migration, and angiogenesis, NFAT-1 mediates both the induced anergy of CD4^+^ T cells through the expression of different inflammatory cytokines and the Treg-mediated suppression of T-helper (Th) cells activation [123,124,125]. Thus, PlGF, in addition to mediating inflammation, could contribute to induce a state of tolerance via NFAT-1, by binding Flt-1/VEGFR-1. Overall, these results further confirm the effects of PlGF on the immune response.

### 2.1. Interplay between Immune Cells and PlGF during Pregnancy

During pregnancy, innate immune cells are the main leukocyte population in the uterus at the time of embryo implantation. Although most studies analyze the role played by a single immune cell type, it is clear that the creation of an adequate microenvironment, supporting the gestation in its various stages, is the result of the reciprocal interaction among mediators and immune cells present during the early stage of pregnancy. In addition to the uNK cells, which are the most abundant and the main protagonists of innate immunity, macrophages and DCs are also present. Importantly, depletion of any of these cell types modifies the uterine environment and hampers the implantation. Changes in the behavior of these cells lead to an imbalance of angiogenic factors and the proinflammatory cytokines and, at the same time, these altered levels of proinflammatory cytokines and angiogenic factors are able to affect the function of immune cells. An altered uterine environment causes defects in trophoblast invasion and placenta damage that trigger a systemic inflammatory response and widespread activation of the endothelium. Thus, the type and function of the immune cells involved in this response are critical and determine whether a viable pregnancy will occur [11,46,126,127].

### 2.2. Natural Killer (NK) Cells

During pregnancy, NK cells are the predominant population of lymphoid immune cells in the uterus at the maternal-fetal interface and are involved both in placental vascular remodeling and in a mother’s immune tolerance [128,129,130,131,132,133,134].

NK cells are members of a rapidly expanding family of innate lymphoid cells (ILCs), derived from early common lymphoid progenitors, the CD34^+^ hematopoietic stem cell [135,136]. Given their heterogeneous characteristics, a new classification that categorizes ILCs into five subsets based on transcription factors and cytokines production has been proposed [137]. These subsets include ILCs1, ILCs2, ILCs3, lymphoid tissue-inducer (LTi) cells, and conventional NK (cNK) cells [138,139]. ILCs interact with the tissue cells to ensure, in addition to the immune response, homeostasis and tissue repair [140,141]. Among these subsets, cNKs are very similar to Tbet^+^ ILCs1 producing IFN-γ but differ in their higher cytotoxic potential. A common feature of cNK cells and tissue resident NK (trNK) cells is their IL-15-dependent signaling during early development, however, they differ in their ability to recirculate [142,143,144]. In fact, cNK cells circulate freely, while trNK cells are resident in the liver, skin, kidney, and virgin uterus [135,136,140,143,144,145].

On the basis of different receptors and transcription factors expression, it is possible to further distinguish cNK cells from trNK cells. In contrast to cNK cells, where CD56 and CD16 expression allows for discrimination of cell cytotoxic and regulatory subsets, uterine trNK cells are almost exclusively CD16^−^CD56^bright^ [146]. In addition, whereas Tbet is required for the development of trNK cells in the liver and skin, uterine trNK develop independently of the Tbet transcription factor [147]. All these differences indicate that cNK and trNK cells in the uterus represent different lineages of NK cells rather than different differentiation states [130,140,145].

In mice, the onset of decidualization is characterized by a series of events such as the extracellular matrix (ECM) remodeling of the endometrial stroma, the induction of angiogenesis, and a significant increase in uNK cells, which originates mainly from local proliferating trNK cells, [73,143,144,148,149,150] while the recruitment of cNK cells takes place later [144,151].

Unlike cytotoxic T lymphocytes, NK cells can eliminate tumor cells, infected cells or nonself cells, by direct contact without a previous activation, due to their natural cytotoxic activity. Direct contact between NK and the target cells may engage activating or inhibitory receptors expressed on NK cells. Each NK cell can express simultaneously several different activating or inhibitory receptors, resulting in the potential for many specificities. The uNK cells cytotoxic ability is regulated by an education process where only those cells that recognize the “self” are promoted to have cytotoxic ability, become tolerant, and act when there is a “dangerous self” or “missing self” signals [152,153]. This ability of NK cells is tightly regulated by a complex of interactions among the target cell and activating or inhibitory receptors expressed on the NK cell surface. If the strength of the activating signals outweighs the inhibitory signals, the cell releases cytolytic granules directed against the target cell and produces cytokines [154,155]. Among these receptors, the expression of killer immunoglobulin-like receptors (KIRs) confers to uNK cells an important function, by inhibiting the production of cytotoxic cytokines and stimulating the production of angiogenic factors [75,131,156]. Thus, resting uNK cells have a low cytotoxic ability as compared with “primed” uNK cells. Additionally, uNK cells priming is regulated by the microenvironment in which the NK cell is present and can also be regulated by the proximity of other immune cell types such as monocytes, DCs, and T cells [157,158,159,160,161].

Genetic association studies have indicated that both uNK cell-activating receptors, KIR2DS4 and KIR2DS1, recognize fetal HLA-A ligands and protect from PE [157,162]. Conversely, pregnant women with a specific KIR haplotype and fetal HLA-C2 genotype combination have a significantly higher risk of PE [163]. Thus, uNK cells can respond to fetal MHC class I via their inhibitory and activating receptors to control appropriate placental vascularization and development.

During the first trimester of pregnancy, uNK cells represent as much as 50–70% of decidual infiltrating lymphocytes and are characterized by CD56^bright^CD16^−^KIR^+^CD9^+^ phenotype [146,164,165]. Unlike cNK cells, uNK cells are poorly cytolytic; they release cytokines/chemokines that regulate the immune environment and angiogenic growth factors, such as PlGF, for placentation [73,129,148,149,150,156,166,167]. One of the plausible mechanisms by which uNK cells are inhibited in their cytotoxic activity is through hypoxia, a condition that is able to stimulate angiogenesis and which is normally present during decidualization. Indeed, it was shown that the cytolytic capacities of uNK cells were markedly and significantly impaired under hypoxic conditions and this inhibition was associated with the activation of transcriptional factor STAT3 [168]. Of interest, as mentioned above, hypoxia upregulates the expression of PlGF, its receptor Flt-1/VEGFR-1 and co-receptor NRP1 [68,69,82].

A further mechanism by which uNK cells induce maternal tolerance is through the crosstalk between uNK and CD14^+^ myelomonocytic cells. These cells are in close contact in the decidua and their interaction is mediated by IFN-γ. Following interaction with uNK cells, decidual CD14^+^ cells express indoleamine 2,3-dioxygenase (IDO) resulting in the induction of Treg cells and immunosuppression [169]. In addition, decidual CD14^+^ cells may also induce Treg cells through transforming growth factor-β (TGF-β) production or Cytotoxic T-Lymphocyte Antigen (CTLA)-4-mediated interactions. Notably, only the interaction between uNK and decidual CD14^+^ cells results in Treg cell induction, whereas cNK or CD14^+^ cells isolated from peripheral blood are ineffective [169]. Therefore, uNK cells, in addition to being involved in the regulation of invading trophoblastic cells and in providing immunity during pregnancy, play an essential role in modulating maternal tolerance [7,128,132,152].

**Figure 2:**
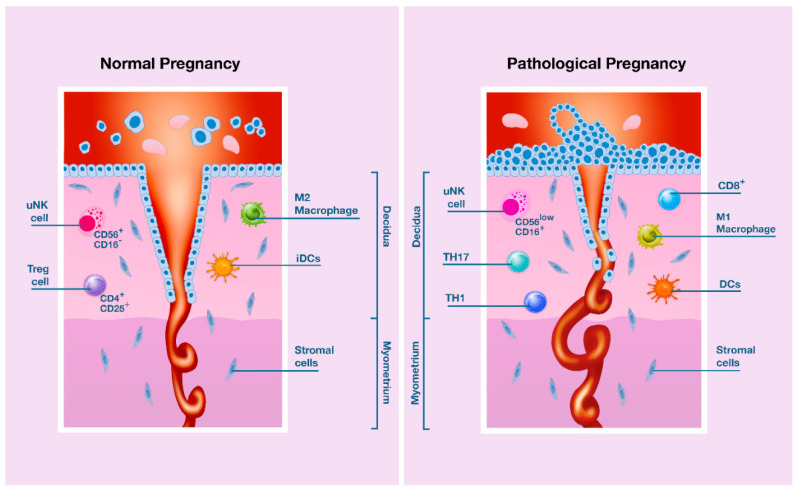
Reduced invasiveness and remodeling of maternal spiral arteries by trophoblastic cells is associated with a risk of pathological pregnancy.

Although the details of signaling pathway triggering cytokines production are still elusive, IFN-γ and angiogenic growth factors such as PlGF produced by uNK cells contribute to spiral arteriole remodeling by acting on endothelial cells and decidual stromal cells [73,129,148,159,170]. By contrast, genetic evidence in humans and mice suggests that excessive inhibition of uNK cells function impedes both decidual arterial remodeling and fetal growth [75,148,167,170,171,172] (Figure 2). However, it has been reported that uNK cells depletion did not reproduce a deficiency in uterine spiral artery remodeling but was associated with a marked maternal uterine vasculopathy at a later time point in gestation in a rat model of PE induced by antibody-based NK cells depletion [171]. This evidence could account for the onset of endothelial dysfunction, and subsequently, PE in late pregnancy.

During a normal pregnancy (Figure 2, left panel) with adequate PlGF levels, EVT cells migrate to the myometrium and infiltrate the endothelium of the maternal spiral arteries. This results in dilatation and increased flow of maternal blood at low pressure into the intervillous space. Uterine NK cells (CD56^+^CD16^−^) and M2 macrophages facilitate deep invasion of trophoblast cells into the myometrium. Moreover, tolerogenic immature dendritic cells (iDCs) promote Treg lymphocytes. Conversely, in pathological pregnancies (Figure 2, right panel) with unbalanced levels of PlGF, the immune cells in the uterine microenvironment (uNK CD56^low^CD16^+^ cells, M1 macrophages, mature DCs, Th1 and Th17 lymphocytes, and CD8^+^ cells) fuel an excessive inflammatory response and reduce the invasion of the trophoblast with less remodeling of the spiral artery. Blood flows at higher pressure resulting in placental stress and reduced placental development.

Uterine NK cells have also been reported to contribute directly to fetal growth by producing growth-promoting factors essential for embryo development prior to the development of the placenta [173]. They also produce several cytokines that recruit and regulate cells of the adaptive immune system as well [161,174]. In addition to the production of PlGF and cytokines, an additional mechanism by which uNK could induce maternal-fetal tolerance appears to promote the induction of Treg cells and suppress Th17 cells [175,176]. Indeed, PE is associated with a reduction in Treg cells in the circulation and decidua and the severity of the disease is related to their reduced number [177]. It is plausible to assume that one of the mechanisms by which uNK cells induce maternal tolerance is through the production of an adequate level of PlGF. In fact, as previously mentioned, the involvement of Flt-1/VEGFR-1 and NRP1 receptors induces the secretion of IL-10 by T lymphocytes [88] and the delayed maturation of DCs [93], respectively, thus, favoring the development of Treg cells.

Of note, serum IL-17 levels have been shown to be significantly higher in PE patients than in healthy non-pregnant and pregnant women, and elevated IL-17 and sFlt-1/PlGF ratio serum level had an additive effect on the risk of PE [178]. Predictably, Th17 cells are higher in PE than in non-PE pregnancy, resulting in an imbalance between Treg/Th17 with a proinflammatory phenotype and an increased secretion of inflammatory cytokines [176]. This evidence has been recently confirmed by Yoo et al. Indeed, Th17 cells selectively secrete PlGF, and PlGF in turn, specifically induces the differentiation of inflammatory Th17 cells by activating the transcription factor STAT3 via binding to Flt-1/VEGFR-1. Moreover, PlGF replaces the activity of IL-6 in the production of IL-17, suppressing the generation of Treg cells [37]. Remarkably, as mentioned above, the hypoxic microenvironment reduces the killing capacity of uNK cells via STAT3 and, at the same time, upregulates PlGF production by uNK cells. By contrast, PlGF secreted by Th17 cells suppresses the generation of Treg cells via STAT3 and induces proinflammatory IL-17 secretion. Overall, these results suggest a refined regulation of the inflammatory response in the early stages of pregnancy, confirming the important role of PlGF in modulating the vascular development of the placenta, and also the behavior of the immune cells.

### 2.3. Macrophages

Macrophages are the second largest group of cells in the decidua and they comprise 20–30% of all leukocytes with CD14^+^CD206^+^ phenotype [179]. Macrophages are characterized by functional plasticity, and therefore their activation can be proinflammatory or anti-inflammatory. They can be classified as “classic” and “alternative” or M1 and M2, respectively. M1 macrophages, by triggering Th1 adaptive immune reactions, have inflammatory and antimicrobial properties and promote the destruction of tissue cells, while M2 macrophages have anti-inflammatory properties and contribute to tissue remodeling, angiogenesis, and wound healing [180,181,182]. The balance of polarization between M1 and M2 macrophages is important for different processes of normal pregnancy, such as trophoblast invasion, spiral artery remodeling, and apoptotic cell phagocytosis [183,184]. In normal pregnancy, many evidences have shown that uterine macrophages (uMφ) have an immunosuppressive phenotype, therefore, M2 immunosuppressive macrophages are necessary for normal pregnancy to also maintain fetal-maternal tolerance [185,186,187].

Although the ratio of M1/M2 macrophages changes during different gestation phases to protect the fetus from the maternal immune microenvironment, some studies have reported that PE could be associated with either a decrease or increase in the number of uMφ [8,185,188]. However, it appears that their phenotype is more important than their number and it is influenced by soluble factors in the microenvironment [182,187]. Moreover, the recruitment of macrophages is a receptor-dependent process and is largely regulated by chemotactic factors and by hypoxia [189,190,191]. In this regard, one of the main chemotactic factors capable of recruiting and polarizing macrophages is precisely the PlGF [82,189]. Additionally, as well as being a survival factor for macrophages, the binding of PlGF to VEGFR-1 promotes and stimulates activation (e.g., cytokine production) of macrophages [33,86]. Although much of the knowledge about PlGF-induced M2 macrophages polarization has been derived from evidence in tumor models, different in vitro and in vivo studies have proven that PlGF polarized macrophages to the M2 phenotype, which in turn, was characterized by PlGF upregulation [83,192]. In fact, in a laryngeal carcinoma model, PlGF-induced M2 polarization was associated with an increase in the expression level of MMP-9 through the activation of TGF-β, which in turn upregulated the PlGF [193]. Moreover, as reported in tumor models, hypoxia strongly increased macrophage-mediated T-cell suppression in vivo, in a HIF-1α macrophage expression-dependent manner [194,195]. Indeed, in addition to being involved in the process of ECM remodeling, angiogenesis, and invasiveness, MMP-9 has been reported to mediate immunosuppression of CD8^+^ lymphocytes through a proteolytic process of IL-2Rα [196]. These results further confirm that human placental development is based on features of tumorigenesis, such as invasiveness, angiogenesis, and evasion of the immune response (Figure 1). Thus, uMφs regulate vascular remodeling by secreting PlGF, VEGF, MMP-9, and MMP-2, enabling trophoblast invasion, and their number is correlated with the expression levels of these angiogenic growth factors in the endometrium. Furthermore, the M2 macrophage phenotype has a higher angiogenic potential than other macrophage subsets, as has been shown in C57BL/6 J mice [197]. However, as macrophages migrate and accumulate in the most hypoxic regions, severe hypoxic (1–3% O_2_) exposure significantly suppressed PlGF expression by M2c subset macrophages as compared with physiological hypoxia (5% O_2_) [190,191]. Of note, M2c macrophages were the cells that produced the highest levels of PlGF [198].

A further link between macrophage polarization and PlGF has been attributed to histidine-rich glycoprotein (HRG). HRG is a heparin-binding plasma protein produced in the liver with anti-inflammatory effects and also synthesized by monocytes and macrophages [199]. HRG is transported as either a free protein or stored in α-granules of platelets and released after thrombin stimulation, and it modulates several functions, including coagulation, immune response, and vascularization, by binding to different cells such as endothelial cells, T cells, and macrophages. HRG shows both antiangiogenic and pro-angiogenic activity depending on the components of the microenvironment or on proteolytic cleavage of the antiangiogenic fragment of HRG [200]. For its features, HRG is also involved in the hypercoagulability and the angiogenic imbalance seen in early-onset PE [201,202]. Furthermore, specific HRG polymorphisms have been associated with recurrent miscarriage [203]. Given its role in angiogenesis, HRG could be responsible for an inadequate implantation and placentation [202,204].

In tumor models, HRG upregulation downregulated M2 markers such as IL-10, CCL22, and PlGF, while simultaneously increasing M1 markers such as IL-6 and CXCL9. Therefore, the reduced expression of IL-10 and CCL22 decreased the recruitment of Treg cells and, consequently, improved the function of DC and T cells and promoted the infiltration of CD8^+^ T cells and NK cells. The mechanism by which HRG influenced M2 polarization was largely due to the downregulation of PlGF [192].

Whether HRG-mediated mechanisms in the tumor microenvironment could play a positive role in the immune response against cancer cells, how they influence a pregnancy remains to be elucidated. In this regard, the answer arises from several studies which have shown that HRG altered levels or polymorphisms were associated with the onset of PE [202,203]. Indeed, levels of HRG decreased during pregnancy in all women, but the levels were significantly lower in women who later developed PE than in normal pregnant women [202]. This finding has been partly explained by authors through the relative hypoxia during early pregnancy in women with PE due to defective placentation because of inappropriate trophoblast invasion of the maternal spiral arteries [202]. Alternatively, it could instead be explained by the onset of PE due to an inadequate amount of PlGF, therefore finely tuned PlGF levels in normal pregnancy are necessary to ensure adequate levels of HRG.

Reasonably, uMφ dysregulated polarization has been associated with inadequate remodeling of the uterine vessels and defective trophoblast invasion, and finally has led to spontaneous abortion, PE, and preterm birth [184,185,186,187,191]. In this regard, Li et al. reported that a pregnancy-induced hypertension (PIH) patient group exhibited a significantly higher percentage of CD86^+^ cells (M1) and a significant lower percentage of CD163^+^ cells (M2), representing a higher M1/M2 ratio than a control group. Moreover, the PIH group expressed higher concentrations of TNF-α and IL-1β, and expressed lower concentrations of IL-4, IL-10, and IL-13 than the control group, indicating a Th1 polarization [205]. It was clear that the functional maturation of macrophages was impaired in patients with PE and that a proinflammatory imbalance with a predominance of the M1 phenotype would be present. This finding was corroborated by an increase in proinflammatory cytokines (such as TNF-α, IL-6, and IL-8) and a decrease in anti-inflammatory cytokines (such as IL-10) in the placenta of preeclamptic patients [13,205,206,207].

uMφs also play a role in controlling local maternal immune responses because they are involved in a crosstalk with NK cells by secreting active TGF-β, which in turn inhibits NK cell effector functions [169,208]. Indeed, after complete placental development, uMφs shift toward a predominantly M2 phenotype, which promotes maternal immune tolerance and protects fetal growth until parturition [8]. Finally, Mφs also have an essential role in adaptive immunity through induction of T cells recruitment and activation and by B cells interaction. Therefore, placental-derived macrophage colony-stimulating factor (M-CSF) and IL-10 induce macrophages to produce IL-10 and CCL18, but not IL-12 or IL-23, thus, driving the expansion of CD25^+^Foxp3^+^ Treg cells in parallel with increased IL-10 production [8,169,209,210,211].

### 2.4. Dendritic Cells

DCs are the third cell group of innate immune cells present in the decidua. DCs have a key role in triggering the immune response by inducing the activation and differentiation of naïve T cells and simultaneously play a critical role for the development of tolerance [212]. The biological plasticity of DCs in promoting immunity or tolerance appears to be dependent on both their maturation state and the microenvironment in which they dwell. Thus, immature DCs are specifically efficient in antigen uptake and presentation, but the low expression levels of MHC gene products and T cell costimulatory molecules may contribute to peripheral tolerance under homeostatic conditions [213].

Concerning the uterine microenvironment, various stimuli such as soluble mediators (e.g., hormones, cytokines, and growth factors) or interactions with other immune cells modulate the tolerogenic activity of DCs, indicating that this behavior is context dependent. It has been reported that estrogen inhibited the ability of DCs to stimulate T cell proliferation and the production of both Th1⁄Th2 cytokines by upregulating IDO that supported the maturation of DCs with tolerogenic properties in rodents [214].

A recent review that focused on the pleiotropic nature of IFN-γ could help clarify the role played by DCs during early pregnancy, a phase characterized by inflammation [215]. Although IFN-γ exhibits extensive proinflammatory effects and has been associated with pathological pregnancies, it can also paradoxically exert an immunosuppressive role on both innate and adaptive immune cell types, promoting DC tolerance through the induction of IDO expression. IFN-γ induced IDO competence is not limited to immune cells but extends to other cell types such as epithelial cells, mesenchymal stem cells, and tumor cells. Therefore, the depletion of tryptophan leads to the inhibition of the effector T cells and promotes the differentiation of the FoxP3^+^ Treg cells [215]. In addition, uMφ may also be an additional source of IFN-γ [216], although uMφ has been reported to suppress IFN-γ production by T cells costimulatory B7-H1/PD-1 signaling during early pregnancy. Therefore, B7-H1, expressed by uMφ, functions as a key regulator of local IFN-γ production, and thus contributes to the development of appropriate maternal immune responses [217]. Several studies have shown that when DCs were stimulated by IFN-γ in the absence of danger or pathogen-related signals, its effects induced predominantly tolerogenic features [218]. In this scenario, the crosstalk between decidua and immune cells contributes to the adequate vascular remodeling of the spiral arterioles and to the protection of the fetus from infections, and also guarantees the development of the tolerogenic microenvironment.

Uterine DCs (uDCs) appear to also have a role in implantation and decidua formation because the presence of CD11c^+^ DCs is critical during early placentation, as the number of uDCs increases at the implantation period and the depletion of these cells leads to implantation failure in mouse models [219,220].

The close contact between uDCs and uNK cells in decidua suggests that there are important reciprocal interactions between them in shaping the decidualizing microenvironment during the early stages of pregnancy [169]. Indeed, uDCs appear to promote the differentiation of uNK cells, because uNK cell functions are impaired in association with decreased levels of IL-15 and IL-12 in implantation sites depleted of uDCs [159,221]. Therefore, the depletion of uDCs, as well as the altered uNK cells maturation, impair tissue remodeling and angiogenesis [221,222,223,224]. In addition, studies have shown that uterine cell proliferation was dependent on a synergistic effect between uDCs and uNK cells, confirming that uDCs-uNK cells crosstalk may be important for the endometrial stroma differentiation during implantation [160,166,169].

Implant failure was also observed in combinations of allogeneic and syngeneic mating after DCs depletion, as DCs depletion altered decidua response [219]. Again, the expression of sFlt-1/sVEGFR-1 in mature human monocyte-derived dendritic cells counteracted their angiogenic properties, and thus prevented adequate implantation [223]. However, since uDCs are generally in an immature state, their role in implantation, on the one hand, is likely redundant and potentially compensated by other immune cells. On the other hand, they likely are more important in playing an early key role in promoting tolerance to paternal antigens. Indeed, altered levels of Treg cells and DCs have been demonstrated in the peripheral blood of women with PE [222,224].

Concerning the PlGF role in inducing tolerance, it has been reported that PlGF inhibited the activation and maturation of human DCs differentiated from CD14^+^ monocytes, effectively and rapidly through the NF-κB signaling pathway [225], confirming the immunosuppressive effect shown by angiogenic growth factors [226,227]. PlGF-treated DCs resulted in the downregulation of the expression of maturation markers CD80, CD86, CD83, CD40, and MHC-II expression, as well as the inhibition of IL-12, IL-8, and TNF-α production in response to LPS stimulation, with respect to untreated DCs. PlGF inhibited DCs maturation through the VEGFR-1, and this PlGF-induced DCs dysfunction was rescued by anti-human Flt-1/VEGFR-1 mAb. Additionally, the treatment of DCs with PlGF resulted in the suppression of naïve CD4^+^ T cell proliferation in an allogeneic mixed lymphocyte reaction. The results from this study indicated that PlGF could downregulate Th1 immune responses by modulating the function of DCs [225].

Last but not least, PlGF is able to recruit bone marrow-derived myelomonocytic cells through its receptor Flt-1/VEGFR-1 via NFAT-1 transcriptional activation [34,228]. These myeloid-derived cells have been shown to exert immunomodulatory effects on the immune cells, especially DCs. Common features of bone marrow-derived myelomonocytic cells are the immature state and a remarkable ability to suppress T cell responses [226,227]. In addition to their suppressive effects on adaptive immune responses, they have also been reported to regulate innate immune responses by modulating cytokines production by macrophages such as IL-6, IL-10, CCL-22, and TGF-β. These cytokines, in turn, promote the differentiation of monocytes to mature macrophages but block their differentiation to DC, decrease the expression of DCs maturation markers, attenuate the Th1 immune response, as well as impair the activity of cytotoxic T lymphocytes and NK cells.

## 3. Bridging PlGF and Hypertension

In recent years, in addition to the known altered mechanisms involved in inducing hypertension (e.g., salt-water balance, cardiovascular function, and peripheral vascular resistance), several clinical and experimental evidences have supported the involvement of the immune system in the occurrence of hypertension [47,48,49,229,230,231,232,233]. This belief arises from observations on animal models, in which immune cells are crucial players in the onset of hypertension, infiltrating vessel walls and kidneys of hypertensive animals [234,235]. However, only a few studies have provided a mechanistic explanation for how immune cell functions promote blood pressure increases. Severe combined immunodeficiency mice or recombinase-activating gene-1 (*Rag-1^−/−^*) knockout mice, which lacked both T and B lymphocytes, showed blunted hypertension responses and did not develop abnormalities of vascular function to angiotensin II (ANGII) treatment [236,237]. However, hypertension and vascular dysfunction were restored when *Rag-1^−/−^* mice received adoptive transfer of T cells, but not B cells. Furthermore, after the adoptive transfer of T cells, *Rag-1^−/−^* mice challenged with ANGII significantly increased T cell production of IFN-γ and TNF-α and treatment with a TNF-α antagonist prevented hypertension induced by ANGII, indicating the role of inflammation in inducing hypertension [237]. In addition, IL-10 knockout mice, unable to direct T cells to an anti-inflammatory phenotype, developed symptoms similar to PE when pregnant, confirming the role of T cells in the onset of this syndrome [238]. Conversely, IL-10 treatment reduced inflammation, endothelial dysfunction, and blood pressure in hypertensive pregnant rats [239]. Again, the evidence indicates that production of proinflammatory cytokines, including TNF-α, IL-17, and IL-6, contribute to hypertension, likely by promoting vasoconstriction, production of reactive oxygen species, and sodium reabsorption in the kidney [240,241]. Remarkably, each of these cytokines is reciprocally interconnected with PlGF because, by activating the same transcription factors, they create a self-feeding circuit [34,36]. Overall, these observations support the role of PlGF in the immune system and also the development of PE.

Experimental evidence in pregnant animals indicates that PlGF is a potent arterial vasodilator and may participate in the mechanisms regulating the maternal vascular tone during pregnancy [242]. Therefore, an inadequate level of PlGF during pregnancy may be accountable for the onset of PE [51,59,77,104,243]. A recent study revealed that PlGF had biological effects on samples of uterine arteries, obtained from normotensive women undergoing cesarean hysterectomy, when challenged with ANGII. This study showed that PlGF contributed to the blunted vascular response to ANGII during normotensive pregnancies and sFlt-1/sVEGFR-1 appeared to attenuate this effect contributing to the regulation of vascular tone by altering the vascular response to ANGII [244].

Although the role of PlGF in inducing hypertension during pregnancy has been associated with several mechanisms involving immune cells and cytokines dysregulation, a definitive link between PlGF-induced hypertension and the immune system, even in non-pregnant animal models, was reported in a study by Carnevale et al. The authors demonstrated that PlGF, through mediating a neuroimmune interaction, played a key role in the spleen’s immune system by organizing the T-cell response to hypertensive challenge in ANGII-treated mice. Whereas the chronic ANGII infusion produced a progressive increase in blood pressure in wild-type (wt) mice, due to an accumulation of T lymphocytes in the aortic wall and kidney, the hypertensive response was completely abolished in *plgf^−/−^* mice. Mechanistically, PlGF mediated the hypertension response to ANGII challenge by repressing tissue inhibitor of metalloproteinases (Timp)-3 protein expression in macrophages, through the transcriptional Sirtuin (Sirt)1-p53 axis. In turn, Timp3 repression allowed the costimulation of T cells and their deployment toward classical organs involved in hypertension [245]. Overall, these data are definitive evidence of the link between PlGF and hypertension through immune-mediated mechanisms.

## 4. Conclusions

The most common conditions encountered during pathological pregnancy are hypertensive complications, including PE, PIH, and maternal chronic hypertension. PE is a systemic syndrome affecting about 5–8% of all pregnancies. It is characterized by new onset of hypertension and proteinuria after 20 weeks of gestation and is a major cause of maternal mortality (15–20% in developed countries) and one of the major causes of infant morbidity and mortality, perinatal deaths, preterm birth, and intrauterine growth restriction [246,247,248].

PE may be the result of abnormal activation of the maternal immune system characterized by endothelial dysfunction and excessive inflammation [57,112,113,206,239,241]. Knowledge regarding the involvement of the immune system in both hypertension and in pregnancies complicated by PE has evolved significantly. During pregnancy, it was believed that alterations in the levels of angiogenic/anti-angiogenic factors such as PlGF and sFlt-1/sVEGFR-1 were essentially responsible for impaired vascular placental development, increased vascular resistance of spiral arteries, and endothelial dysfunction. Today, we know that these factors also have a decisive role in modulating the maternal immune response throughout the pregnancy. They are not only accountable for alterations of immune cell behavior but also for the altered release of proinflammatory mediators and systemic involvement of the inflammatory response.

PlGF showed an important effect on the development of hypertension by modulating egress of T cells from the spleen and their accumulation in vessel walls and kidneys, even in non-pregnant mouse model [245]. It is not surprisingly that its influence is modulated by the involvement of typical pathways implicated in tumor growth. Indeed, some of the effects attributed to PlGF inhibition in cancer might rely on epigenetic modulation of the p53-Timp3 axis, which is well known to also play a crucial role in tumor growth [249], again confirming the presence of overlapping mechanisms between pregnancy and malignancy.

Presently, the diagnostic criteria of PE remain uncertain because no known specific biomarkers are available, thus, women at risk are identified based on epidemiological and clinical risk factors [250]. Many studies on PE diagnosis, which have analyzed the sFlt-1/sVEGFR-1/PlGF ratio as a predictor for poor pregnancy, have demonstrated its reliability [100,102,103,104]. However, PlGF imbalance during pregnancy can be due to several mechanisms, which preclude the precise identification of a specific marker useful for diagnosis. In this regard, different scenarios can be expected. On the one hand, PlGF upregulation can be accountable for the activation of an inflammatory state associated with the release of mediators such as TNF-α, IL-6, IL-17, and COX-2. These cytokines, by modulating the cells of the immune system (e.g., M1 polarized Mφ, mature DCs, Th1 lymphocytes, reduction of Treg cells) could prevent maternal tolerance, fuel inflammation, and, at the same time, interfere with adequate vascular development of the placenta. Both of these events contribute to mechanisms that promote hypertension. On the other hand, reduced levels of PlGF, due either to inadequate production by trophoblast cells, uterine cells and uNK cells, or to its excessive sequestration by sFlt1/sVEGFR-1, can be responsible for the inadequate trophoblast invasion, the deficient vascular development and, at the same time, for a loss of maternal tolerance. Yet again, these events contribute to mechanisms that promote hypertension. Therefore, to date, a single reliable diagnostic marker is still difficult to identify. In the future, the definitive understanding of the immunological mechanisms involved in the onset of different PE phenotypes, such as placental PE associated with growth restriction, PE associated with a chronic maternal inflammatory condition, and maternal dysmetabolism associated with normal fetal growth, in which PlGF could be involved, may offer new specific diagnostic markers and therapeutic tools for managing pathological pregnancies.

## Footnote

Figure 1 was modified by Holtan et al. [19] with kind permission by Publisher, License Number: 4911830648497.
